# Occupational noise effects on hearing thresholds, speech perception, and self-reported hearing abilities in young adult dental hospital personnel

**DOI:** 10.2478/aiht-2026-77-4131

**Published:** 2026-09-25

**Authors:** Büşra Terzioğlu, Büşra Türkoğlu, Ömer Faruk Demir, Ali Cemal Yumuşakhuylu, Berceste Güler Ayyıldız

**Affiliations:** Kütahya Health Sciences University, Tavşanlı Vocational School of Health Services, Dental Services Programme, Kütahya, Turkey; Kütahya Health Sciences University, Oral and Dental Health Application and Research Centre, Kütahya, Turkey; Marmara University Institute of Health Sciences, Department of Audiology and Speech Disorders, Istanbul, Turkey; Kütahya Health Sciences University, Tavşanlı Vocational School of Health Services, Audiometry Programme, Kütahya, Turkey; Marmara University Faculty of Medicine, Department of Otorhinolaryngology, Istanbul, Turkey; Kütahya Health Sciences University Faculty of Dentistry, Department of Periodontology, Kütahya, Turkey

**Keywords:** audiometry, dental service, occupational exposure, signal-to-noise ratio, speech in noise hearing test, Speech, Spatial and Qualities of Hearing Scale, dentalna služba, izloženost na radu, omjer signala i buke, slušni test govora u buci, tonska audiometrija, *Speech, Spatial and Qualities of Hearing Scale*

## Abstract

This cross-sectional study aimed to evaluate the effects of occupational noise exposure on objective auditory function and self-reported hearing difficulties in young adult dental professionals. It included 56 young adults (aged 18–40), divided into the noise-exposed (n=31) and unexposed control group (n=25). Auditory assessments consisted of pure-tone audiometry (250–8000 Hz), word recognition in clinical speech-in-noise (SIN) test at 0 dB and −5 dB signal-to-noise ratios, and the self-reported Speech, Spatial and Qualities of Hearing Scale (SSQ). No significant differences were observed between the exposed and control groups in pure-tone thresholds or SIN performance, but the exposed group reported significantly poorer hearing abilities on the SSQ Speech (7.53, 8.30; p=0.043) and SSQ Qualities (8.30, 8.91; p=0.014) subscales. These findings suggest that standard clinical assessments may overlook early functional deficits associated with occupational noise and that self-reported hearing difficulties may complement conventional audiometric assessment before measurable threshold shifts occur.

## Abstract (hr)

Cilj ovoga presječnog istraživanja bio je procijeniti učinke profesionalne izloženosti buci na objektivnu slušnu funkciju i subjektivno procijenjene teškoće sa sluhom u mladih dentalnih radnika. Istraživanje je obuhvatilo 56 sudionika u dobi od 18 do 40 godina, koji su bili podijeljeni u skupinu izloženu buci (n=31) i neizloženu kontrolnu skupinu (n=25). Procjena sluha obuhvaćala je tonsku audiometriju (250 – 8000 Hz), prepoznavanje riječi u kliničkom testu govora u buci (eng. s*peech-in-noise*, krat. SIN) pri omjerima signala i buke od 0 dB i −5 dB te upitnik *Speech, Spatial and Qualities of Hearing Scale* (SSQ) za subjektivnu procjenu sluha. Između skupine izložene buci i kontrolne skupine nisu utvrđene statistički značajne razlike u pragovima sluha izmjerenima tonskom audiometrijom, kao ni u rezultatima SIN testa, ali je skupina izložena buci na SSQ-u iskazala statistički značajno slabije slušne sposobnosti u podljestvicama *Speech* (7,53; 8,30; p=0,043) i *Qualities* (8,30; 8,91; p=0,014). Ovi rezultati upućuju na to da standardne kliničke procjene mogu previdjeti rane funkcionalne poremećaje povezane s profesionalnom izloženošću buci te da subjektivno procijenjene teškoće sa sluhom mogu nadopuniti konvencionalnu audiometrijsku procjenu prije pojave mjerljivih promjena pragova sluha.

According to the US National Institute on Deafness and Other Communication Disorders (NIDCD) and the Occupational Safety and Health Administration (OSHA), prolonged exposure to noise above 85 A-weighted decibels (dBA) as an 8-hour time-weighted average can cause irreversible damage to the human ear (1, 2). Noise-induced hearing loss (NIHL) occurs when sensitive structures in the inner ear are damaged (3).

Noise may also affect the ability to understand speech when background sounds are present (4), such as those in dental practice acoustic settings, in which devices produce sounds at various frequencies at the same time. Previous studies reported that noise levels produced by dental equipment (high- and low-speed handpieces, ultrasonic scalers, suction units, amalgamators) range from 60 to 99 dBA (5, 6). What also adds to the background noise are air conditioning systems, human voices, and the building's acoustic structure (7). NIHL in dental professionals typically begins in the 3–6 kHz frequency range (acoustic notch) (8, 9).

However, a significant discrepancy is often observed between standard audiometric thresholds and dental professionals' specific auditory complaints, such as difficulty understanding speech in noise, even when their audiograms are within "normal" limits (<25 dB) (10,11,12). In fact, recent evidence suggests that traditional pure-tone audiometry may not capture the full extent of noise-induced auditory damage, as long-term noise exposure can selectively damage the synaptic connections between inner hair cells and auditory nerve fibres long before permanent threshold shifts occur (13). This condition is known as cochlear synaptopathy or hidden hearing loss (14). In such subclinical stages, individuals frequently experience heightened listening effort and real-world communication difficulties that conventional clinical tests miss (15, 16). To address this discrepancy, objective tests are now being complemented with the Speech, Spatial and Qualities of Hearing Scale (SSQ) as a comprehensive tool for measuring auditory disabilities in real-life settings (17).

Furthermore, literature consistently reports a positive correlation between NIHL and age, particularly in 40-year-olds and over, which introduces presbycusis as a confounding factor that complicates the isolation of occupational noise effects from natural ageing (8, 9, 18).

To address these issues, we decided to take a comprehensive approach that would encompass objective and subjective measures of hearing impairment by comparing them between occupationally noise-exposed clinical and laboratory dental staff and unexposed administrative personnel working in the same dental hospital. To minimise the potential confounding effect of ageing, the study was limited to young adults aged 18–40 years.

Our null hypothesis was that there would be no statistically significant differences between the occupationally exposed and unexposed personnel in any of the hearing impairment measures.

## PARTICIPANTS AND METHODS

This cross-sectional, controlled study was carried out at the Audiometry Application Laboratory of the Kütahya Health Sciences University, Tavşanlı Vocational School of Health Services, between August and October 2025 and was approved by the Non-Interventional Clinical Research Ethics Committee of the Kütahya Health Sciences University (approval No. 2025/06-30) and conducted in accordance with the Declaration of Helsinki. Personal data of all participants were anonymised, and each participant signed an informed consent form. The study was designed following the Strengthening the Reporting of Observational Studies in Epidemiology (STROBE) guidelines (19).

### Participants

The sample included employees at the Tavşanlı Oral and Dental Health Centre. Participants were divided into two groups according to their routine occupational exposure to noise generated by dental equipment. For this study, we defined occupational noise as the noise generated by high- and low-speed handpieces, ultrasonic scalers, suction systems, and dental laboratory equipment commonly used in dental practice.

Candidates for the exposed group included dentists, dental assistants, and dental technicians who routinely worked with these noise-generating devices and reported occupational noise exposure. All were full-time employees working approximately eight-hour workdays, five days a week.

Candidates for the control group included administrative staff, cleaning personnel, security staff, and technical service workers whose routine duties were primarily office- or non-clinical-based, did not involve the use of noise-generating dental equipment, and who reported no exposure to occupational noise in their work environment.

Sample size, calculated with G*Power 3.1.9.2 (Heinrich Heine University Düsseldorf, Düsseldorf, Germany), was set to a minimum of 21 participants per group, assuming test power of 80 % (1 − β=0.80), significance level of α=0.05, and a large effect size (Cohen's d=0.80). Furthermore, we aimed to include all available dental staff who met the inclusion criteria to improve the sample's representativeness. The inclusion criteria were 1) having answered all demographic and SSQ questions; 2) age 18–40 years; 3) consenting to participate in the study; and 4) having a Type A tympanogram finding. From the total of 72 candidates screened for eligibility we excluded two from the prospective exposed group (one due to pre-existing low-frequency hearing loss and one due to profound high-frequency hearing loss) and 14 from the prospective control group (seven due to being ≥40 years of age, three due to a history of significant non-occupational or recreational noise exposure, two due to ototoxic medication use, one due to profound high-frequency hearing loss, and one due to the presence of a tympanostomy tube). The remaining 56 participants – 31 in the exposed group and 25 in the control group – had clear institutional health records and no medical history of any intellectual disability or cognitive impairment.

### Objective and subjective measurements of hearing

Pure-tone audiometry was conducted using a Harp Inventis audiometer (Inventis, Padova, Italy) to determine hearing thresholds at 250 Hz to 8000 Hz in accordance with the established clinical standards (20).

To evaluate auditory discrimination ability, we ran the standard Word Recognition Test (WRT) using isophonically balanced monosyllabic word lists developed for adults (21). This method was selected because it is routinely available in clinical audiology practice and can readily be applied in occupational hearing assessments. The researcher presented the stimuli via a monitored live voice, calibrated to peak at 0 volume units (VU). The assessment was conducted in a sound-treated booth to ensure a controlled environment.

Subsequently, to evaluate speech-in-noise (SIN) recognition, the test was repeated with the introduction of ipsilateral speech noise at signal-to-noise ratios (SNR) of 0 dB and −5 dB as described elsewhere (22). The SIN scores were recorded as the percentage of correctly identified monosyllabic words and a higher SIN percentage indicated better speech perception performance in noise.

To evaluate self-reported auditory performance in real-life scenarios, we relied on the SSQ, which is a scale originally developed by Gatehouse and Noble (17) and adapted for the Turkish population by Kılıc et al. (23) (Cronbach's alpha=0.984). It consists of 32 items across two subscales – Speech (14 items) and Qualities (18 items) – scored using a 0–10 visual analogue scale. Higher scores (closer to 10) indicate better self-perceived auditory performance and higher quality of hearing, whereas lower scores indicate impaired self-perceived hearing.

### Statistical analysis

Primary outcomes consisted of between-group comparisons of the three auditory measures: pure-tone audiometry thresholds, SIN values, and the SSQ Speech and Qualities scores. The secondary outcomes examined the effects of demographics (age, gender, education, and professional experience in years) on hearing thresholds and SIN scores.

Statistical analyses were performed using IBM SPSS Statistics version 26 (IBM Corp., Armonk, NY, USA). Descriptive data are presented as numbers (n), percentages (%), means ± standard deviations (SD), or medians (M) and interquartile ranges (IQR). After confirming the assumption of normal distribution (skewness ≤2.0; kurtosis ≤7.0), we used the independent samples *t*-test for inter-group comparisons of numerical data and Pearson's chi-squared or Fisher's exact tests for the comparison of categorical data. The paired sample *t*-test was used for intra-group comparisons, such as differences in right and left ear measurements, and effect sizes were calculated using Cohen's d. The relationships between the variables were analysed with the Pearson correlation coefficient. Statistical significance was set at p<0.05 for all analyses.

## RESULTS

Statistical analysis revealed no significant differences between the two groups regarding age, gender, education, and years of professional experience. Gender distribution was comparable between the exposed (17 males, 14 females) and control group (16 males, 9 females). The mean age was 28.58±3.83 years for the exposed and 30.64±5.24 years for the control group. Most participants in both groups had a university-level education or higher (90 % in the exposed v 76 % in the control group) and similar mean years of professional experience (6.13±4.45 years v 5.20±3.56 years, respectively).

### Objective hearing results and correlations with age and years of professional experience

There were no statistically significant differences between the exposed and control group in all pure-tone thresholds (from 250 to 8000 Hz) (Table 1) or SIN scores (0 dB and −5 dB SNR) (Table 2) in either the right or left ear. Table 3 shows a significant positive correlation between age and high-frequency pure-tone thresholds, especially at 4000 Hz in the right ear and at 4000 Hz, 6000 Hz, and 8000 Hz in the left ear. A significant negative correlation was observed between age and the 250 Hz threshold in the left ear.

**Table 1:** Comparison of pure-tone thresholds measurements between dental (exposed) and control staff (n=56)

		**Right ear**				**Left ear**			
		**Exposed group (n=31)**	**Control (n=25)**	**Test**	**p**	**Exposed group (n=31)**	**Control (n=25)**	**Test**	**p**
		**Mean±SD**	**Mean±SD**			**Mean±SD**	**Mean±SD**		
Pure-tone thresholds (dB HL)	250 Hz	15.16±3.98	13.8±6.81	0.934	0.355	15.00±3.87	13.6±6.21	1.032	0.307
	500 Hz	13.71±3.15	13.00±5.77	0.585	0.561	14.35±3.09	12.60±6.14	1.389	0.171
	1000 Hz	9.84±3.29	10.40±3.51	−0.616	0.540	10.32±3.40	10.00±4.79	0.294	0.770
	2000 Hz	7.90±4.43	7.80±3.56	0.094	0.925	8.06±4.02	8.80±4.40	−0.653	0.516
	4000 Hz	9.84±4.91	9.20±6.07	0.435	0.665	10.00±7.42	10.60±7.68	−0.296	0.768
	6000 Hz	13.39±7.89	19.20±16.12	−1.765	0.083	12.74±8.45	14.60±12.98	−0.646	0.521
	8000 Hz	15.16±10.12	18.80±17.58	−0.971	0.336	15.16±13.26	18.20±17.55	−0.738	0.464

* p<0.05 (independent samples *t*-test)

**Table 2:** Comparison of SIN measurements between groups and their correlation with age (n=56)

		**Exposed group (n=31)**	**Control (n=25)**	p-value^*^	**Correlation with age (n=56)**	p-value^**^
		**Mean±SD**	**Mean±SD**		**r**	
Right ear	0 dB SNR	67.55±7.79	70.56±7.91	0.159	−0.183	0.178
	−5 dB SNR	44.52±12.88	45.12±12.78	0.862	−0.275^*^	**0.040**
Left ear	0 dB SNR	68.00±8.88	67.52±9.89	0.849	−0.194	0.151
	−5 dB SNR	47.16±13.14	46.56±13.11	0.865	−0.362^*^	**0.006**

SIN – speech-in-noise; SNR – signal-to-noise ratio.

* p<0.05 (independent samples *t*-test);

** Pearson correlation coefficient for age

**Table 3:** Relationships between pure-tone thresholds and age in all participants (n=56)

			**Age**	
			r	p
Pure-tone thresholds (dB HL)	Right ear	250 Hz	−0.258	0.055
		500 Hz	−0.252	0.061
		1000 Hz	−0.079	0.561
		2000 Hz	0.044	0.746
		4000 Hz	0.419^*^	0.001
		6000 Hz	0.259	0.053
		8000 Hz	0.238	0.078
	Left ear	250 Hz	−0.335^*^	0.012
		500 Hz	−0.238	0.077
		1000 Hz	−0.020	0.886
		2000 Hz	0.200	0.140
		4000 Hz	0.521^*^	0.000
		6000 Hz	0.367^*^	0.005
		8000 Hz	0.441^*^	0.001

* p<0.05 (Pearson correlation coefficient)

Regarding SIN performance, age had a significant negative correlation with −5 dB SNR scores in both the right and left ear (Table 2).

As for years of professional experience, Table 4 shows a significant negative correlation at 500 Hz in the left ear and a strong positive correlation at 8000 Hz in the exposed group. In dental staff, years of professional experience also significantly correlated with SIN scores in the right ear for both 0 dB and −5 dB SNR (Table 4).

**Table 4:** Relationships between hearing thresholds, SIN measurements, and years of experience in the exposed group (n=31)

			**Years of experience**	
			**r**	**p**
Pure-tone thresholds (dB HL)	Right ear	250 Hz	0.027	0.885
		500 Hz	−0.107	0.568
		1000 Hz	0.036	0.849
		2000 Hz	0.183	0.323
		4000 Hz	0.352	0.052
		6000 Hz	0.020	0.913
		8000 Hz	0.059	0.754
	Left ear	250 Hz	−0.275	0.134
		500 Hz	−0.377^*^	0.036
		1000 Hz	−0.068	0.717
		2000 Hz	−0.212	0.253
		4000 Hz	0.041	0.826
		6000 Hz	0.033	0.860
		8000 Hz	0.531^*^	0.002
SIN (%)	Right ear	0 dB SNR	0.447^*^	0.012
		−5 dB SNR	0.370^*^	0.041
	Left ear	0 dB SNR	−0.047	0.801
		−5 dB SNR	−0.216	0.243

SIN – speech-in-noise; SNR – signal-to-noise ratio.

* p<0.05 (Pearson correlation coefficient)

To further examine whether the observed association between years of professional experience and SIN performance was independent of education and age influences, we ran partial Pearson correlation analysis, which showed that the previously significant correlations for the right ear were no longer significant, regardless of SNR. The same happened when both education and age were controlled simultaneously (Table 5).

**Table 5:** Correlations between years of professional experience and SIN performance in the exposed group (n=31)

	**Years of experience**							
			**Zero-order Pearson**		**Partial Pearson (education controlled)**		**Partial Pearson (education and age controlled)**	
			**r**	**p**	**r^2^**	**p**	**r^2^**	**p**
**SIN %**	Right ear	0 dB SNR	0.447^*^	0.012	0.120	0.526	0.138	0.474
		−5 dB SNR	0.370^*^	0.041	0.156	0.412	0.193	0.316
	Left ear	0 dB SNR	−0.047	0.801	−0.007	0.973	−0.018	0.928
		−5 dB SNR	−0.216	0.243	0.130	0.493	0.133	0.490

SIN – speech-in-noise; SNR – signal-to-noise ratio.

* p<0.05 (Pearson correlation coefficient)

### SSQ Speech and Qualities subscale results

Table 6 shows that the exposed dental staff had significantly lower SSQ Speech and Qualities scores than control (p=0.043 and p=0.014, respectively).

**Table 6:** Comparison of SSQ scores between dental (exposed) and control staff (n=56)

	**Exposed group (n=31)**	**Control (n=25)**	**Total (n=56)**	**Test**	**p**	**Effect size**
SSQ Speech Score (Mean±SD)	7.53±1.30	8.30±1.48	7.87±1.43	−2.071	**0.043** ^*^	−0.557
SSQ Qualities Score (Mean±SD)	8.30±0.91	8.91±0.85	8.57±0.93	−2.545	**0.014** ^*^	−0.684

SSQ – Speech, Spatial and Qualities of Hearing Scale.

* p<0.05 (independent samples *t*-test)

## DISCUSSION

The central finding of our study is that despite no differences between the occupationally noise-exposed young adult dental staff and controls in objective hearing measurements, the SSQ revealed significantly poorer self-reported hearing in the exposed group.

Some studies (9, 24,25,26) reported significantly poorer threshold values in dentists than controls, particularly at higher frequencies, but others (27,28,29), like ours, reported no significant differences in pure-tone thresholds between noise-exposed dental professionals and matched controls. Because all participants in our study were younger than 40, their cumulative noise exposure may not have been long enough to produce clinically measurable threshold shifts. Alternatively, our findings suggest that conventional pure-tone audiometry alone may lack the sensitivity required to detect early auditory changes associated with occupational noise exposure. Previous studies have suggested that noise exposure may affect suprathreshold auditory processing, including neural synchrony, temporal resolution, and central auditory integration, even in the absence of permanent threshold elevation (13, 30, 31).

One possible explanation for the discrepancy between the SIN performance, which revealed no differences between the exposed and control group, and the SSQ findings is that speech-recognition accuracy alone may not entirely reflect hearing issues, as some studies (32, 33) report that SIN relies heavily on cognitive resources such as working memory and attention, which may compensate for peripheral deficits under challenging listening conditions. These, however, we did not measure and compare between the groups. Poorer SSQ Speech and Qualities scores in the noise-exposed group suggest that self-reported hearing measures may capture aspects of everyday listening that are not reflected in SIN scores under controlled clinical conditions. SSQ is particularly sensitive to real-world auditory challenges, as it reflects speech understanding in dynamic, spatially complex, and cognitively demanding environments (17). Moreover, Gatehouse and Noble (17) emphasise that SSQ outcomes often diverge from audiometric thresholds and may reveal functional listening deficits despite normal hearing levels. In fact, similar discrepancies between SIN performance and self-reported hearing difficulties have been reported earlier by Couth et al. (34) and Smith et al. (35), indicating that clinical SIN measures and subjective questionnaires may provide complementary information rather than assessing identical aspects of auditory function. Future studies incorporating objective or subjective measures may help clarify the mechanisms underlying these findings.

### Correlations with age, education, and years of professional experience

The correlation between age and NIHL associated with dental environment noise is already well known (36,37,38,39). Our study, however, has revealed that at high-frequency pure-tone thresholds (4–8 kHz) such correlation exists even in the younger population below 40.

Furthermore, in the exposed group of dental workers, the 8 kHz frequency threshold in the left ear also correlates with years of professional experience, which is in line with some earlier reports (26, 40). This points to a cumulative effect of occupational noise exposure, even if it does not cause clinically significant loss, especially in the left ear, as indicated elsewhere (27, 41, 42).

Similarly, the significant negative correlation between SIN scores and age indicates that subclinical auditory changes may begin early and remain within normal audiometric thresholds. In our study, years of professional experience positively correlated with the SIN scores in the right ear of the exposed dental workers at both 0 dB and −5 dB SNR conditions (Table 5), initially suggesting improved speech recognition performance with increasing professional experience. However, when education level was introduced as a control variable in partial correlation analyses, this association became non-significant, which is consistent with the concept of cognitive reserve positing that individuals with higher levels of education are better able to utilise cognitive resources such as working memory, attention, and executive functions to compensate for challenging listening conditions (43, 44). SIN relies not only on peripheral audibility but also on top-down mechanisms that support lexical access and contextual restoration (45, 46).

### Study limitations

Although we recruited all eligible personnel who met the inclusion criteria to maximise the available sample, its size remains small, which may limit the statistical power to detect subtle differences in pure-tone thresholds. Another limitation is the absence of *in-situ* noise dosimetry, which would have provided a more objective assessment of individual occupational noise exposure (47), nor did we measure exposure to dental chemicals (such as acrylic monomers frequently used by dental technicians) for potential synergistic effects with noise (48).

While focusing on a young adult cohort may have minimised the confounding cumulative effects of ageing and lifelong environmental noise exposure, the observed associations between age, years of professional experience, and SIN performance should be interpreted with caution, as subtle age-related auditory changes and sample heterogeneity cannot be completely excluded. Additionally, the observed discrepancy between standard audiometric thresholds and lower SSQ scores in our study may point to recall bias among the exposed dental workers, which introduces a priming effect during self-reporting (49). Comprehensive central auditory processing (CAP) assessments could therefore provide deeper insights by looking beyond peripheral hearing using electrophysiological tools such as Auditory Brainstem Response (ABR) or cortical evoked potentials (13). Such methods may help in exploring potential subclinical synaptopathy or subtle central processing changes that are not typically captured by conventional pure-tone audiometry. The inclusion of more sensitive objective measures, such as Extended High-Frequency (EHF) audiometry (50) or Otoacoustic Emissions (OAEs) (51), could further illuminate the earliest stages of cochlear damage.

Overall, incorporating these aspects into longitudinal studies could support the development of more refined hearing conservation strategies and ergonomic recommendations tailored to the unique dental hospital environment.

## CONCLUSION

Our findings indicate lower subjective hearing quality and speech communication scores (measured by the SSQ) in young adult dental staff exposed to occupational noise than in unexposed controls, even though pure-tone thresholds and objective SIN performance remain comparable. This discrepancy may point to early auditory issues, emerging before measurable deficits can be captured by standard objective audiometric tests. Positive correlations between high-frequency hearing thresholds and age and years of professional experience suggest a cumulative effect on auditory sensitivity. Our findings support the integration of subjective assessment tools into occupational screening test batteries to aid in early detection of potential functional auditory difficulties in dental professionals. Future studies, preferably longitudinal by design, should include larger samples and advanced suprathreshold measures to determine whether these early subjective symptoms eventually progress to clinically evident noise-induced hearing loss.
